# Update in Autoimmune Movement Disorders: Newly Described Antigen Targets in Autoimmune and Paraneoplastic Cerebellar Ataxia

**DOI:** 10.3389/fneur.2021.683048

**Published:** 2021-08-18

**Authors:** Madeline Garza, Amanda L. Piquet

**Affiliations:** Department of Neurology, University of Colorado, Aurora, CO, United States

**Keywords:** autoimmune cerebellar ataxia, paraneoplastic cerebellar ataxia, AP3B2 antibody, ITPR1 antibody, TRIM9 antibody, TRIM67 antibody, TRIM46 antibody, mGluR2 antibody

## Abstract

Movement disorders are a common feature of many antibody-associated neurological disorders. In fact, cerebellar ataxia is one of the most common manifestations of autoimmune neurological diseases. Some of the first autoantibodies identified against antigen targets include anti-neuronal nuclear antibody type 1 (ANNA-1 or anti-Hu) and Purkinje cell cytoplasmic antibody (PCA-1) also known as anti-Yo have been identified in paraneoplastic cerebellar degeneration. Historically these antibodies have been associated with an underlying malignancy; however, recently discovered antibodies can occur in the absence of cancer as well, resulting in the clinical syndrome of autoimmune cerebellar ataxia. The pace of discovery of new antibodies associated with autoimmune or paraneoplastic cerebellar ataxia has increased rapidly over the last few years, and pathogenesis and potential treatment options remains to be explored. Here we will review the literature on recently discovered antibodies associated with autoimmune and paraneoplastic cerebellar ataxia including adaptor protein-3B2 (AP3B2); inositol 1,4,5-trisphophate receptor type 1 (ITPR1); tripartite motif-containing (TRIM) proteins 9, 67, and 46; neurochondrin; neuronal intermediate filament light chain (NIF); septin 5; metabotropic glutamate receptor 2 (mGluR2); seizure-related 6 homolog like 2 (SEZ6L2) and homer-3 antibodies. We will review their clinical characteristics, imaging and CSF findings and treatment response. In addition, we will discuss two clinical case examples of autoimmune cerebellar ataxia.

## Introduction

Cerebellar ataxia has a broad differential diagnosis including both acquired and genetic causes, however autoimmune etiologies or paraneoplastic cerebellar degeneration (PCD) should be considered in most case, particularly in adults (1). The first autoantibodies to be identified against neuronal targets include anti-neuronal nuclear antibody type 1 (Anti-Hu) in 1965 (2) and Purkinje cell cytoplasmic antibody (anti-Yo) in 1983 (3) which both represent classic paraneoplastic neurological syndromes. It is hypothesized that cross-reactivity between proteins expressed on the tumor and neuronal antigens is responsible for the development of neurologic symptoms, of which cerebellar ataxia is one of the most common manifestations (4). Since those two autoantibodies were described, the rate of discovery of new autoantibodies to other neuronal targets has been rapidly increasing and there are now several antibodies known to cause cerebellar ataxia both in the setting of malignancy (i.e. paraneoplastic) and in its absence (i.e. idiopathic autoimmune).

Paraneoplastic cerebellar degeneration is a diagnosis under the umbrella of immune-mediated cerebellar ataxias (IMCAs), which also includes post-infectious cerebellitis and gluten ataxia (5). Patients who present with subacute onset cerebellar ataxia but in the absence of malignancy or a known pathogenic antibody are now deemed to have primary autoimmune cerebellar ataxia (PACA) (6). This is in contrast to neuronal antibodies that have already been shown to be directly involved in the pathogenesis of ataxia (e.g. dipeptidyl-peptidase-like-6 [DPPX], metabotropic glutamate receptor 1 [mGluR1], glutamic acid decarboxylase [GAD]-65). See Table 1 for clinical criteria for PACA. These patients tend to present with a subacute onset of gait and limb ataxia as well as dysarthria or nystagmus and they may have objective findings such as inflammatory cerebrospinal fluid (CSF) or magnetic resonance imaging (MRI) showing abnormalities in cerebellum. Patients with suspected PACA should receive immunotherapy trials such as steroids, intravenous immunoglobulins (IVIg) or plasmapheresis (PLEX), although the likelihood of improvement seems to depend on whether the antibody target is a cell surface or an intracellular antigen (5, 6).

**Table 1:** Criteria for the diagnosis of PACA.

1. Predominantly subacute or acute pure cerebellar syndrome (gait ataxia that may be associated with variable degrees of limb incoordination, dysarthria, nystagmus, diplopia) 2. MRI at presentation usually normal or may show primarily cerebellar vermian atrophy with (if available) reduced MR spectroscopy (NAA/Cr ratio) of the vermis 3. At least 2 of the following: a. CSF pleocytosis and/or positive CSF-restricted IgG oligoclonal bands b. History of other autoimmune disorders or family history of autoimmune disorders in first-degree relatives c. Presence of antibodies that support autoimmunity but not yet shown to be either directly involved in ataxia pathogenesis which includes autoantibodies associated with non-neurological autoimmune disease^*^ or autoantibodies reported in only a few patients with ataxia (therefore significance is less well characterized but raises suspicion of PACA)^**^, or to be markers of ataxia with a known trigger^***^ 4. Exclusion of alternative causes made by an experienced neurologist or ataxia specialist (including other causes of immune ataxia such as PCD, GA, PIC and ones that are associated with well-characterized pathogenic antibodies)

* *Examples include thyroid peroxidase, thyroglobulin, anti-SSA (Ro) and anti-SSB (La)*.

** *Examples include anti-ITPR1, anti-Homer-3, anti-AP3B2, anti-neurochondrin, anti-Septin-5, anti-MAG*.

*** *Examples include anti-Yo in paraneoplastic cerebellar degeneration or antigliadin in gluten ataxia. Adopted from: (6). CSF, cerebrospinal fluid; GA, gluten ataxia; MRI, magnetic resonance imaging; NAA/Cr, N-Acetylaspartate/Creatine; PACA, primary autoimmune cerebellar ataxia; PCD, paraneoplastic cerebellar degeneration; PIC, post-infectious cerebellitis*.

The aim of this review article is to focus on recently discovered autoantibodies against neuronal targets identified in syndromes cerebellar ataxia and includes two clinical case examples of adaptor protein-3B2 (AP3B2) and tripartite motif-containing (TRIM)-46. In these the majority cases, an associated antibody has been identified, but it is not yet clear whether there is a direct pathogenic effect with the exception of Metabotropic glutamate receptor-2 (mGluR2).

Table 2 includes an expanded summary on recently described autoantibodies including seen in PACA as well as prominent extracerebellar phenotypes (e.g. Caspr2).

**Table 2:** Summary table of autoantibodies against neuronal targets identified in syndromes of cerebellar ataxia.

**Antibody**	**Average age (range) in years**	**Clinical presentations**	**Diagnostic testing**	**Response to immunotherapy**	**Association with cancer**	**Antigen target location/antibody testing method**	**References**
AP3B2	39 (24–58)	Cerebellar ataxia,Peripheral neuropathy	CSF: pleocytosis, elevated protein, and/or increased IgG index and OCB MRI: cerebellar atrophy	Poor	1/ 9 patients had cancer (RCC)	Intracellular/ IFA staining in cerebellum spinal cord gray matter, dorsal root ganglia and sympathetic ganglia; confirmed with WB and CBA	(7)
Caspr2	50s (19–80); reported in pediatric case series with mean age of 13 (2–17 years)	Encephalitis, Cerebellar ataxia, Morvan Syndrome, peripheral nerve hyperexcitability, neuromyotonia	CSF- About 25% with abnormalities including pleocytosis and/or elevated protein, rarely OCB MRI- About half abnormal with T2 hyperintensities in temporal lobes or hippocampal/mesial temporal atrophy	Positive	SCLC, Thymoma	Extracellular/ IFA; confirmed on CBA^*^	(8–12)
GFAP	42 (21–73)	Meningoencephalitis, myelitis, ataxia and other movement disorders	CSF- Almost all patients with pleocytosis, elevated protein, half with OCB MRI- Abnormal with T2 hyperintensities or contrast enhancement	Positive	Thymoma in about $\frac{1}{4}$, other rare associations (breast, ovarian, GI, prostate, melanoma, parotid adenoma, teratoma)	Intracellular/ IFA with cytoplasmic filament staining restricted to astrocyte populations; confirmed on CBA^*^	(13–16)
Homr 3	Only three case described	Cerebellar ataxia Encephalitis	CSF- pleocytosis, elevated IgG index in one patient MRI- Variable, can be normal or have cerebellar atrophy	Variable	1 SCLC identified, otherwise none	Intracellular	(17–19)
IgLON5	64 (46–83)	Sleep disorder, bulbar symptoms, gait abnormalities, and oculomotor difficulties	CSF- half normal and other half with elevated protein, pleocytosis or OCB MRI- normal in most patients; a subset with mild brainstem and cerebellar atrophy	Poor	None	Extracellular/ IFA; confirmed on CBA^*^	(20)
ITPR1	64 (7–83)	Cerebellar ataxia, Peripheral Neuropathy, Encephalitis, myelopathy	CSF: pleocytosis and/or elevated protein MRI: Varies, can be normal or have T2 signal changes in various locations, cerebellar atrophy also seen	Poor	5/14 patients had cancer (3 breast, 1 RCC, 1 endometrial)	Intracellular/ IFA staining in cerebellum in a “Medusa head-like" cytoplasmic staining pattern; confirmed with CBA^*^	(21, 22)
KLHL11	28.5 (9–76)	Brainstem and cerebellar syndrome, encephalitis	CSF- pleocytosis, elevated protein, increased IgG index and/or OCBs MRI- Mostly abnormal with cerebellar atrophy or midbrain/cerebellar T2 hyperintensities	Variable	Teratomas, Testicular seminoma, mixed germ cell tumors	Intracellular/ IFA with robust reactivity with cytoplasm of large neurons in brainstem and deep cerebellar nuclei; confirmed by CBA	(23, 24)
mGluR2	Only 2 patients, 78yo F and 3yo F	Cerebellar ataxia	CSF only obtained on one patient and was normal MRI- one with increased T2 signal in cerebellum, other with enhancement of cerebellum	Unclear (1 positive, 1 poor)	Small cell tumor of unknown origin and alveolar rhabdo-myosarcoma	Extracellular/ IFA with staining in the cerebellum limited to granular cell layer and hippocampus; confirmed with CBA	(25)
NIF	65 (47–87)	Cerebellar ataxia, encephalopathy, cranial neuropathies	CSF- pleocytosis, elevated protein and/or OCB MRI- Variable, some normal, some with T2 signal or enhancement	Positive	Neuro-endocrine tumors	Intracellular/ IFA with intense staining of neuronal cytoplasmic filaments in CNS and cerebellar granular layer and peri-Purkinje cell regions; confirmed with CBA^*^	(26)
Neuro chondrin	27 (2–67)	Cerebellar ataxia	CSF- all with pleocytosis or OCB MRI- cerebellar and supratentorial gray matter atrophy	Positive	No malignancies identified	Intracellular/ IFA with synaptic-type hippocampal staining pattern; confirmed with WB	(27, 28)
Septin 5	59 (47–62)	Cerebellar ataxia, oscillopsia	CSF- only 1 patient with data available, elevated protein MRI- one normal, one with cerebellar atrophy	Variable	No malignancies identified	Intracellular	(29)
SEZ6L2	62 (54–69)	Cerebellar ataxia, extrapyramidal symptoms	CSF- pleocytosis in 1 patient, others normal MRI- Cerebellar atrophy	Poor	1 patient with ovarian cancer diagnosed 4 years later	Extracellular/ IFA with intense staining of neuropil of hippocampus and molecular layer and synaptic buttons of granular layer of cerebellum; confirmed with CBA	(30–32)
TRIM 46, 9 & 67	71 (64–78)	46-Cerebellar ataxia, 9 & 67- Cerebellar ataxia, RPD, encephalomyelitis	CSF- pleocytosis, elevated protein, OCB MRI- largely normal	Poor	SCLC	Intracellular/ IFA; confirmed with CBA	(33, 34)

* *Antibody commercially available for testing. AP3B2, Adaptor Protein-3B2; Caspr2, contactin-associate protein-2; CBA, cell-based assay; CSF, cerebrospinal fluid; GFAP, glial fibrillary acidic protein; IFA, tissue-based indirect immunofluorescence assay; IgG, immunoglobulin G; ITPR1, Inositol 1,4,5-trisphosphate Receptor Type 1; KLHL11, Kelch-like protein 11; MRI, magnetic resonance imaging; mGluR2, metabotropic glutamate receptor 2; NIF, neuronal intermediate filament light chain OCB, oligoclonal bands; RCC, renal cell cancer; RPD, rapidly progressive dementia; SCLC, small cell lung cancer; SEZ6L2, seizure-related 6 homolog like 2; TRIM, tripartite motif-containing; WB, western blotting assay*.

## Methods

This review focuses on newly described antibodies in the literature seen in PACA and antibody syndromes in which are newly commercially available for testing – thus our understanding of these antibodies will continue to evolve and these syndrome will likely become more readily recognized. We performed a systematic literature search in PubMed to identify autoantibodies reported against neuronal targets in autoimmune and paraneoplastic cerebellar ataxia after 2014. We also included antibody syndromes that are more recently commercially available in the United States for testing since 2021 (35, 36).

### Adaptor Protein-3B2 (AP3B2)

β-neuronal adaptin-like protein, located in neuronal cytoplasm, mediates synaptic vesicle coat assembly. Darnell and colleagues first identified it as an antigen target in 1991 in a 32-year-old woman with subacute progressive ataxia (5). At the time, this was the only known case, but more recently, Honorat and colleagues identified a series of patients with this novel but identical sera and CSF immunofluorescence assay (IFA) staining patterns on mouse nervous system tissue (7). This antigen was identified as β-neuronal adaptin-like protein, now named adaptor protein-3B2 (AP2B2).

All patients reported presented clinically with subacute progressive gait disturbances, including both cerebellar and sensory ataxias. These clinical presentations coincide with the areas of the nervous system that were shown to have greatest staining by IFA; namely the cerebellar Purkinje cells, the dorsal spinal column and dorsal root ganglia. Malignancy in general did not seem to be a precipitant of this autoimmune cerebellar ataxia, with only one patient out of nine found to have an underlying cancer (renal cell carcinoma) (7).

In terms of objective data, CSF was abnormal in all patients. Slight pleocytosis was present in a few patients (median WBC count 7/μL) and others had elevated protein (median 46mg/dL) and increased immunoglobulin-G (IgG) index or oligoclonal bands (7). In the patients who presented with ataxia, brain MRIs showed cerebellar atrophy. Unfortunately, patients treated with immunotherapies did not report improvement, and some were noted to have progressive symptoms despite treatment (7).

Given its intracellular location, AP3B2 antibody is unlikely pathogenic, rather simply a biomarker for CD8+ cytotoxic T cell-mediated damage.

#### Clinical Case Vignette #1

A 15-year-old female with no significant past medical history presented with progressive left sided weakness and ataxia. One month prior, she noticed her left hand became clumsy, her handwriting became illegible (she is left-handed) and she had difficulty completing fine motor tasks such as painting her nails. She also developed a feeling of “heaviness” in her left leg and was unable to keep up with her teammates at practice. With the progression of her symptoms including worsening gait, she was referred to see a neurologist.

**Figure 1:**
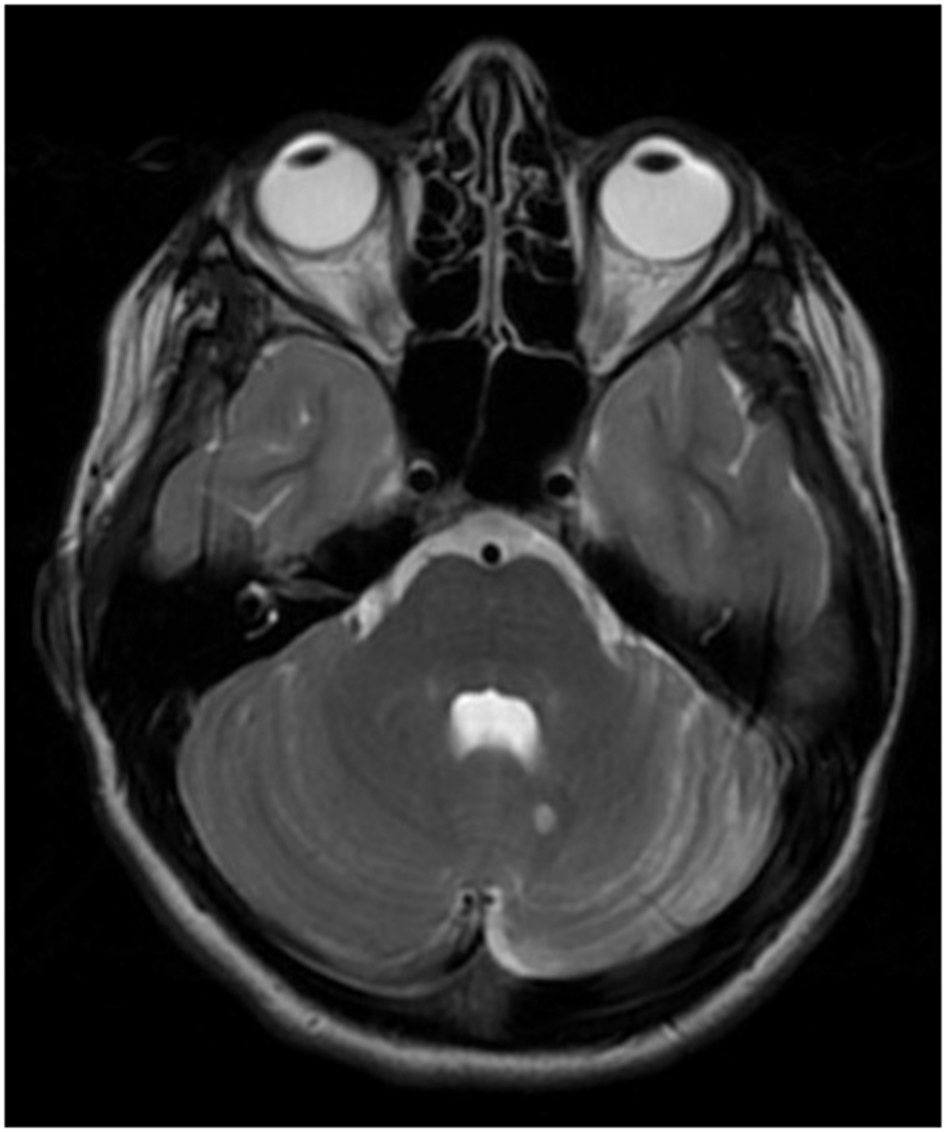
T2 sequence of brain MRI demonstrating hemi-atrophy of the left cerebellum in addition to a 6 mm T2-hyperintense lesion near the cerebellar vermis on the left. Additional brain imaging not show any further T2/FLAIR (fluid-attenuated inversion recovery) abnormalities in the cerebellum, cerebrum or corpus callosum (sequences not shown).

On exam, the patient demonstrated focal left-sided abnormalities including dysmetria with finger-to-nose and heel to shin, dysrhythmia of finger taps and difficulty with rapid alternating movements. Cranial nerves were all intact and there was no dysarthria or truncal ataxia. Brain MRI brain showed mild left cerebellar atrophy as well as a 6 mm T2 hyperintense lesion to the left of the cerebellar vermis (Figure 1). Lumbar puncture revealed inflammatory CSF with WBC 23 (lymphocytic predominance) and 13 unique oligoclonal bands. An extensive infectious workup was unrevealing including Next-Generation sequencing (NGS) (UCSF center for Next-Gen precision for diagnostics). She was started on empiric IV methylprednisolone for treatment of a presumed autoimmune disorder. Positron emission tomography and computed tomography (PET-CT) scan was negative for malignancy, although notably three weeks prior to the onset her neurologic symptoms she did have a pilomatrixoma removed from her neck, which was of unclear significance. Autoantibody testing on her CSF performed at Mayo Clinic Laboratories was positive for an unclassified antibody with synaptic antibody features.

The patient was discharged home after treatment with IV methylprednisolone and was subsequently treated with weekly IV methylprednisolone 1g (as well as one 3 day course of IVIg which she didn't tolerate due to headache) before being started on rituximab at 1,000 mg day 0 and day 14. She responded well to rituximab therapy and has had moderate improvement in coordination on the left and improvement in her gait. She received rituximab therapy over the course of one year and has since remained off immunotherapy with clinical and radiographic follow up over three years. Repeat MRIs have been stable in terms of degree of atrophy in left cerebellum and size of the T2 hyperintense lesion. She is screened yearly for malignancy, which has been negative over the course of three years. Two years after her diagnosis of autoimmune cerebellar ataxia, her CSF evaluation at Mayo Clinic Laboratories revealed a positive AP3B2 antibody. Two unique features seen in this case, not reported in the prior literature, include the hemi-cerebellar atrophy and the positive response to immune therapy.

### Inositol 1,4,5-Trisphosphate Receptor Type 1 (ITPR1)

Inositol 1,4,5-trisphosphate receptor type 1 (ITPR1) is an intracellular ligand-gated calcium channel mainly expressed in membranes surrounding the endoplasmic reticulum. It was first identified as an antibody associated with autoimmune cerebellar ataxia in 2014 by Jarius and colleagues after immunohistochemical testing showed binding of IgG1 antibodies in molecular and Purkinje cell layers on animal cerebellum sections in a pattern similar to, but not matching that of, anti-Ca/anti-RhoGTPase-activating protein 26 (ARHGAP26) antibodies (21, 37).

The largest cohort of patients described with ITPR1-IgG antibodies included five who presented with cerebellar ataxia at an average age of 64 (22). Of the five patients with CSF available, four had abnormalities such as pleocytosis or elevated protein. About half of the patients with ITPR1 IgG antibodies had an underlying malignancy including three breast and one renal carcinoma. Immunotherapy response in these patients is reported to be poor with all ten patients who received immunotherapies in this review failing to improve. Interestingly, there was one case report of a 31-year-old woman with a three-year history of slowly progressive cerebellar ataxia found to have ITPR1 antibodies and BRCA1 mutation. Serial malignancy screenings identified a ductal carcinoma 6 years later, which also had significant ITPR1 expression (38).

The fact that ITPR1 antibodies express such high affinity for Purkinje cells in patients who present with cerebellar ataxia and with such high titers suggests that these antibodies could be pathogenic (37). Additionally, ITPR1 mutations are known to be associated with spinocerebellar ataxias. More studies needed to determine whether ITPR1 may indeed be pathogenic.

### Tripartite Motif-Containing (TRIM) Proteins 9, 67, and 46

Tripartite motif (TRIM) containing proteins are part of a large group of E3 ubiquitin ligases involved in many different processes such as cellular signaling, carcinogenesis and immunity. TRIM 9 and TRIM 67 have a large role in neuronal development and axonal growth and are highly concentrated in Purkinje cells both in the hippocampus and cerebellum (33). TRIM 46, expressed more diffusely in the central and peripheral nervous system, plays a role in axon growth (34). All three of these specific TRIM proteins have been described as antigen targets in a handful of patients presenting with paraneoplastic cerebellar ataxia.

The few patients described with TRIM9 and TRIM67 have all presented with a subacute onset of severe cerebellar ataxia (33). Presentations with TRIM 46 antibodies seem to be more varied (which could be explained by more diffuse expression of TRIM 46 in the CNS) and has included encephalitis and rapidly progressive dementia, along with cerebellar ataxia (34). Brain imaging is reported as normal in most of these patients or if abnormal may demonstrate some cerebellar atrophy. CSF is often inflammatory with pleocytosis, elevated protein or the presence of oligoclonal bands. These antibodies are strongly associated with malignancy, specifically small-cell lung cancer (33, 34). In the report of two patients with anti-TRIM9 and anti-TRIM67 antibodies, one patient treated with immunotherapy did not show any improvement, and one other patient who had regression of his cancer continued to have severe ataxia years down the line (33). This finding suggests that TRIM9 and TRIM67 are biomarkers of rare cases of paraneoplastic cerebellar ataxia and prompt diagnosis is necessary to try to initiate early immunotherapy, as the mechanism of damage is likely related to CD8+ T cells that seems to cause irreversible neuronal death (33).

#### Clinical Case Vignette #2

**Figure 2:**
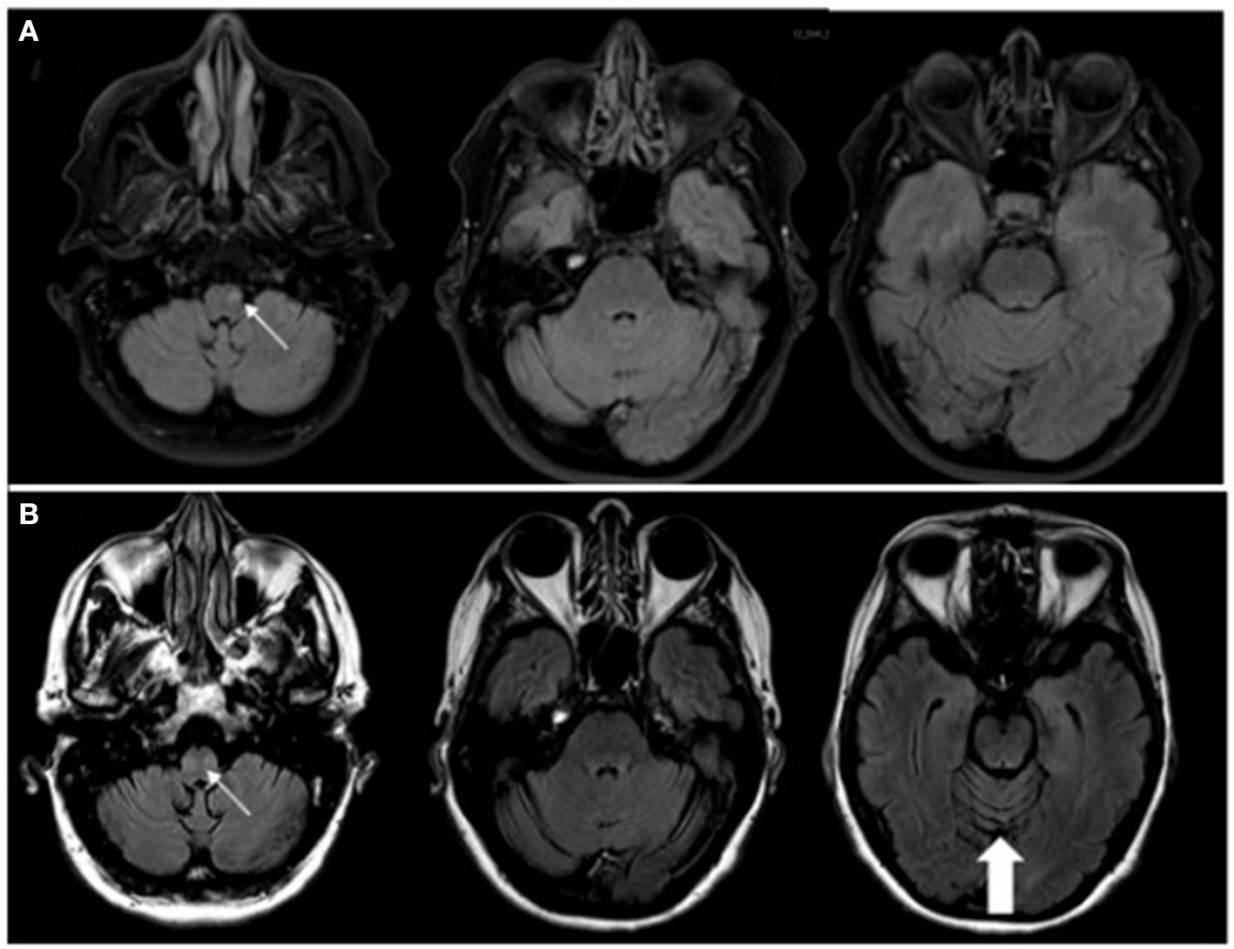
**(A)** MRI sequences of T2/FLAIR (fluid-attenuated inversion recovery) with STIR (short tau inversion recovery; i.e. fat suppression) at the onset of neurological symptoms of vertigo and ataxia demonstrating a small T2, non-enhancing lesion (contrast imaging not shown) in the left medulla (thin, white arrow). **(B)** with T2/FLAIR MRI 3 months later demonstrating T2 changes seen bilaterally within the medulla (thin, white arrow) with no associated contrast enhancement. There is noted cerebellar atrophy, particularly seen in the superior cerebellum (thick, white arrow), compared to the MRI from 3 months prior.

**Figure 3:**
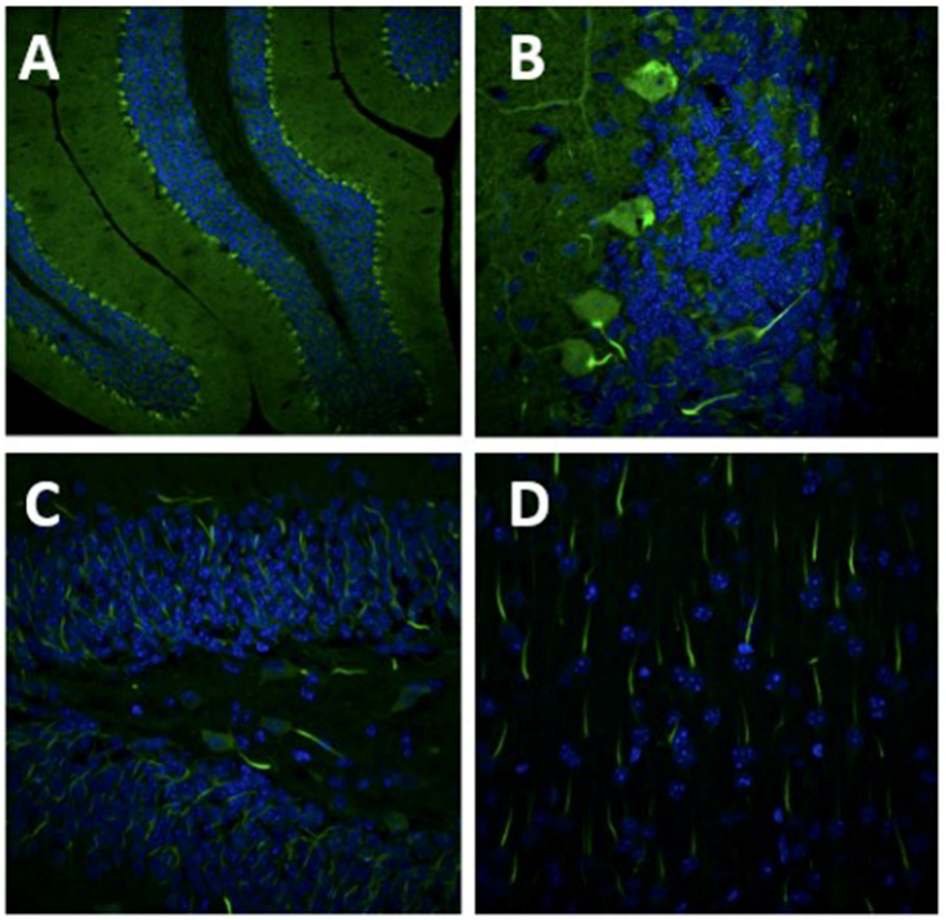
Images of **(A)** 10x magnification and **(B)** 60x magnification of cerebellar tissue demonstrating antigen staining of the patient's Trim46 antibody (in green) and nuclei are in blue. **(C)** There are axon initial segments staining in green in the dentate gyrus and **(D)** in the cortex as well. *Images courtesy of Dr. Christopher Bartley and team at University of San Francisco (USCF) Weill Institute for Neurosciences laboratory*.

A 63-year-old woman with a one-year history of metastatic endometrial cancer was recently started on pembrolizumab and lenvatinib for treatment of her cancer. Seven months after initiation of pembrolizumab she developed neurological symptoms of vertigo and ataxia. Pembrolizumab was discontinued and she was started on a short course of high-dose oral steroids with no response in her neurological symptoms. She continued to progress with worsening mobility, gait imbalance, diplopia and vertigo with nausea and vomiting so she was referred to Neurology for further evaluation. Brain MRI at the time of her symptom onset was only remarkable for a small T2 lesion the left medulla and no evidence of cerebellar atrophy. Repeat brain MRI at the time of her neurological evaluation revealed subsequent cerebellar atrophy, worsening T2 signal change in the medulla bilaterally (Figure 2), and new T2/FLAIR signal change in the left thalamus. CSF analysis revealed 0 WBCs, mildly elevated protein at 67 mg/dL (ref <45 mg/dL), 6 unique oligoclonal bands, elevated IgG index of 1.27 (ref 0.28–0.66 ratio), and an unclassified antibody identified on IFA screen at Mayo Clinic Laboratories. Subsequent research antibody testing revealed a positive TRIM46 antibody confirmed on cell-based assay (CBA). Novel autoantibody testing completed on a research basis at UCSF laboratories also confirmed the presence of TRIM46-IgG (Figure 3). The patient was subsequently diagnosed with anti-TRIM46 antibody-associated cerebellar ataxia with suspected paraneoplastic etiology vs. pembrolizumab-associated autoimmune cerebellar ataxia. Notably, immune checkpoint inhibitors (ICIs) can cause a variety of clinical phenotypes and various underlying disease mechanisms have been observed. When classic paraneoplastic associations are seen (i.e. classic phenotypes such as cerebellar ataxia with clear antibody profiles associated with cancer), this could potentially be augmented by the anticancer immune response against onconeural antigens (39). This patient will remain off her pembrolizumab given this complication. She received acute treatment with 5 days of IV methylprednisolone and plasma exchange followed by the initiation of monthly IV cyclophosphamide. Two months after her treatment with immune therapy, she demonstrated mild improvement in her mobility (she is able to walk and transfer with assistance after being wheelchair-bound) and improvement of her vertigo. However, at six months after completion of her cyclophosphamide course she remains severely disabled neurologically.

### Neurochondrin

Neurochondrin is an intracellular protein expressed in neurons in a somato-dendritic distribution. In concert with G-protein-coupled receptors such as mGluR1 and mGluR5, it has been shown to have an important role in synaptic plasticity, particularly in the cerebellum (27).

There have been two small cohorts of patients described that were found to have anti-neurochondrin antibodies. They all presented with a rapidly progressive cerebellar ataxia, and a few demonstrated brainstem findings such as eye movement abnormalities or dysphagia. CSF had an inflammatory profile in all patients, including pleocytosis, elevated protein, oligoclonal bands and/or increased IgG index. While some MRIs were initially normal or showed increased T2/FLAIR signal (mainly in pons, midbrain, and hippocampus), almost all patients eventually developed significant cerebellar atrophy on subsequent MRI scans (27, 28). Malignancy was only identified in one patient (uterine carcinoma) (28). Unfortunately, none of the patients treated with immunotherapy showed any signs of improvement.

Given its intracellular expression, damage related to anti-neurochondrin IgG antibodies is likely mediated by a cytotoxic T-cell response.

### Neuronal Intermediate Filament Light Chain

Neuronal intermediate filaments (NIF) are a group of proteins that are integral to the structure and function of neurons in the central and peripheral nervous systems. Neurofilament light chain proteins are a subtype of NIF proteins that have been implicated as antigen targets in patients with cerebellar ataxia and encephalopathy (26).

Subacute but rapidly progressive cerebellar ataxia as well as encephalopathy were the two most common clinical presentations in a large cohort of patients described in 2018 (26). Of the 9 patients with data available, 5 had an abnormal MRI consisting of cerebellar atrophy and/or T2 hyperintensities in patients with ataxia (26). Most patients had CSF abnormalities including lymphocytic pleocytosis (median 41.5 WBCs in patients with CA) and/or CSF-restricted oligoclonal bands and increased protein (median 116 mg/dL) (26). NIF-IgG was strongly associated with malignancy, with cancer in 77% of these patients, most commonly neuroendocrine tumors including small-cell lung cancer. Patients with NIF antibodies with reported immunotherapy treatment tended to have improvement of their neurologic symptoms, which is less common for paraneoplastic neurologic disorders mediated by antibodies to intracellular antigens (26).

### Septin 5

Septins are a group of cytoskeletal GTP-binding proteins with many different functions; although in the CNS they seem to have an important role in synaptic vesicle formation and exocytosis (40). The anti-Septin 5 antibody was identified by Honorat and colleagues at Mayo Clinic when indirect IFA of patient's sera and CSF demonstrated a novel staining pattern of synaptic regions of mouse cerebrum and cerebellum (29).

Anti-septin 5 antibodies have been identified in only a handful of patients. Clinically these patients all presented with a rapidly progressive cerebellar syndrome, including two with oscillopsia. CSF was only available for one patient and was significant for increase IgG synthesis rate. MRI imaging was only available for two patients, one of which was normal and the other showing cerebellar atrophy. There were no malignancies identified in any of the patients. In terms of treatment, only two patients were treated with immunotherapies which resulted in significant improvement for one patient, but only transient improvement in the other patient who ended up dying 6 months later (29).

Although septin-5 is largely expressed intracellularly, it is revealed extracellularly during exocytosis. It is therefore unclear whether septin-5 antibodies directly pathogenic or if they mediate damage via cytotoxic T-cells.

### Metabotropic Glutamate Receptor 2 (mGluR2)

Metabotropic glutamate receptors (mGluR) are a family of cell surface G-protein-coupled receptors that bind glutamate and therefore play a significant role in synaptic transmission and neuronal excitability. Metabotropic glutamate receptor 1 was the first to be identified in patients with cerebellar ataxia in the early 2000s. More recently in 2019, mGluR2 was described in two patients with subacute onset cerebellar ataxia (25). There are 8 subtypes of mGluRs divided into 3 subgroups: Group I includes mGluR1 and 5 in which both are autoimmune targets in cerebellar ataxia and encephalitis associated with Hodgkin lymphoma (41, 42); Group II comprises of mGluR2 and 3; and Group III includes the remaining subtypes. The main physiologic role of mGluR2 is to modulate glutamatergic and GABAergic synaptic transmission and mGluR2 antibodies could potentially alter these functions (25, 43). Immunohistochemical studies in rat cerebellar cortex demonstrated mGluR2 and 3 immunoreactivity in the cerebellar cortex localizing to the Golgi cells with the majority of the Golgi cells distributed mainly in the Purkinje cell layer and superficial part of the granular layer (44).

The two patients described with anti-mGluR2 antibodies are on opposite ends of the spectrum in terms of age; one patient was a 78-year-old woman and the other a 3-year-old girl. The older woman presented initially with intermittent episodes of ataxia that eventually became progressive and constant. The young girl developed ataxia and dysarthria after a 3-day prodrome of fever, nausea and vomiting. Both patient's MRIs were abnormal with hyperintense T2 cerebellar lesions including patchy enhancement of cerebellum in the 3-year-old. CSF was unavailable for the older patient but was normal in the young girl. Both received immunotherapy with drastically different outcomes. The older woman had progressive symptoms unresponsive to therapies while the young girl had complete recovery after receiving IV methylprednisolone and IVIg. Malignancy was identified in both patients after the development of neurologic symptoms. Small-cell neuroendocrine cancer of unknown origin was discovered on inguinal node biopsy in the 78 year-old woman three years after symptom onset, and the young girl was found to have alveolar rhabdomyosarcoma one year later (25).

Unlike other paraneoplastic cerebellar ataxias described here, mGluR2 antibodies target an extracellular antigen. Like anti-mGluR1 antibodies, mGluR2 antibodies likely have a direct pathogenic effect.

### Seizure-Related 6 Homolog Like 2 (SEZ6L2)

SEZ6L2 is highly expressed in the hippocampus and cerebellar cortex and is an auxiliary subunit of the α-Amino-3-hydroxy-5-methyl-4-isoxazolepropionic acid (AMPA) receptor (30). SEZ6L2 modulates AMPA receptor function by binding to adducin and glutamate receptor 1 (45). In 2014, SEZ6L2 antibodies were identified in a patient with subacute cerebellar ataxia and retinopathy, and later in another patient with cerebellar ataxia associated with hypomimia, bradykinesia, and postural instability (30–32). Since then, Landa and colleagues described four more cases in 2020 with SEZ6L2 antibodies identified in serum and CSF by immunohistochemistry on rate brain sections and immunoprecipitation from rat cerebellar neurons; all patients were then identified on CBA with unclassified neuropil antibodies (32). The median age of these four patients was 62 years old and patients presented with a subacute gait ataxia, dysarthria and mild extrapyramidal symptoms (32). Only one out of four patients had a CSF pleocytosis and three patients had evidence of moderate cerebellar atrophy with no evidence of contrast enhancement or other inflammatory features (32). In these series, none of the patients improved with immunotherapy (32).

### Homer-3

Homer-3 is expressed at a high level in Purkinje cells (17, 37). Anti-Homer-3 antibody-associated cerebellar ataxia is rare and only reported in three cases (17–19, 37). The first case was presented in a 65-year-old female with vertigo, vomiting, dysarthria and severe subacute limb and gait ataxia (18). A second case was then reported in a 38-year-old man with a pancerebellar syndrome followed by encephalitis, seizures, and papilledema (19). Later a third case was described in a 51-year-old women with a pancerebellar syndrome (17). CSF analysis was abnormal in all cases including lymphocytic pleocytosis in two and oligoclonal bands in the last case. Follow up MRI was available in two patients, both reporting cerebellar atrophy. The 65-year-old woman had no improvement with steroids, while the other two patients were reported to have a partial improvement with immunotherapy which included a combination of steroids, IVIg, and mycophenolate mofetil (MMF) (17–19, 37).

### Other Antibodies Associated With Cerebellar Ataxia

There are a handful of other autoantibodies that are associated with symptoms of cerebellar ataxia, although clinical manifestation of these antibody syndromes predominately present with extracerebellar phenotypes (Table 2). IgLON5 has a unique presentation consisting mainly of sleep disorders, bulbar symptoms and gait abnormalities (not necessarily all attributed to cerebellar dysfunction). Cerebellar ataxia in anti-IgLON5 disease is rare, however it has been described along with MRI findings of cerebellar atrophy (20). In anti-contactin-associated protein-like 2(Caspr2) antibody syndrome, unique symptoms such as peripheral nerve hyperexcitability and neuromyotonia tend to accompany the encephalitis and ataxia (8, 46). In a cohort of 37 patients with anti-Caspr2 antibodies (20 with encephalitis and 17 with neuromyotonia), 5 patients with transient symptoms suggestive of cerebellar impairment suggesting that cerebellar ataxia could be observed in up to 25% of patients (9). Kelch-like protein 11 (KLHL11) have been identified as a biomarker of a paraneoplastic brainstem cerebellar syndrome associated with testicular seminoma (23). While often presenting with cerebellar ataxia, more recently expanded phenotypes in KLHL11 have been described including co-existing anti-NMDAR encephalitis, brainstem diencephalic encephalitis, opsoclous-myoclonus, limbic and extralimbic encephalitis and chronic psychosis in the setting of teratomas (24). Autoimmune glial fibrillary acidic protein (GFAP) astrocytopathy is a newly described autoimmune meningoencephalomyelitis syndrome associated with ataxia in 40% of reported cases (13).

In terms of paraclinical data, the majority of patients with GFAP and KLHL11 antibodies had an inflammatory CSF profile (including pleocytosis, elevated protein, OCBs and/or elevated IgG index), while only about half of patients with IgLON5 and a quarter of patients with Caspr2 antibodies had these same abnormalities. Cerebellar atrophy was one of the more common MRI findings along with T2/FLAIR signal changes in the cerebellum, brainstem, or hippocampi. In GFAP astrocytopathy the majority of patients have characteristic T1 postgadolinium enhancement including patterns of radial periventricular, leptomeningeal and punctate, and serpiginous and periependymal enhancement; spinal cord enhancement can also be seen often with a central cord pattern (13).

## Discussion

Here we have reviewed recently described autoantibodies associated with cerebellar ataxia including anti-AP3B2, anti-ITPR1, anti-TRIM, anti-neurochondrin, anti-NIF, anti-septin 5,anti-mGluR2, anti-SEZ6L2, and anti-homer 3. Clinically, patients present with a subacute to rapidly progressive cerebellar ataxia. The majority of cases reported an inflammatory CSF profile with a lymphocytic pleocytosis and/or the presence of unique oligoclonal bands. Imaging abnormalities are more variable but when present often involve T2-hyperintensities or atrophy of the cerebellum.

Main differences between the antibodies described lies in their associations with malignancy and reported responses to treatment. Malignancy was less common in patients with anti-AP3B2 antibody-associated cerebellar ataxia with only 1 in 9 patients identified as having an underlying cancer. On the other hand, anti-NIF antibodies are strongly associated with neuroendocrine tumors and the few patients described with anti-TRIM antibodies were all found to have lung adenocarcinomas. ITPR1 antibodies seem to be associated with breast cancer although reports of malignancy types were more variable. Both patients with mGluR2 antibodies had malignancies of different types. No malignancies have been reported with anti-neurochondrin and with anti-SEZ6L2 only 1 patient was identified with a ovarian cancer, diagnosed 4 years later. In contrast, KLHL11 has a strong association with testicular seminoma in men.

Most of the described antibodies in this review, except for mGluR2 and SEZ6L2 (see details in Table 2), are directed against intracellular antigens. It is widely hypothesized, that antibodies against intracellular targets are not directly pathogenic and rather mediate damage via cytotoxic T-cells, although there has been some evidence to dispute this assumption. For example, studies have shown that neurons are able to take up IgG containing anti-Yo, which subsequently resulted in Purkinje cell death (39). The same direct cytotoxic effect has also been found with anti-Hu antibodies (27), although it remains unclear exactly how these antibodies cause destruction of neurons. Regardless of whether the antibodies themselves or cytotoxic T-cells are mediating the damage, these conditions are often reported to be less responsive to immunotherapies. This appears to hold true of the newly discovered antibodies described in this review with the only exception being anti-NIF antibody cerebellar ataxias, where most patients had improvement of symptoms with immunotherapy. Furthermore, while SEZ6L2 is an extracellular antibody, the immunotherapy response in the 4 reported patients was poor.

In general, autoimmune and paraneoplastic cerebellar ataxias result in progressive and debilitating symptoms, therefore, it is crucial for clinicians to be aware of this diagnosis so treatment can be started as early as possible, ideally before significant, irreversible neuronal cell death has taken place. Any patient presenting with a subacute onset of cerebellar ataxia without a family history of genetic ataxia, an autoimmune pathogenesis should be considered. The absence of an antibody does not exclude neurological autoimmunity and if suspected immunotherapy should be considered, especially if the diagnostic work up reveals an inflammatory CSF profile. Equally important, these patients need screening for underlying malignancy as the neurologic syndrome is often the first indication of a neoplasm. There remains much to learn regarding pathogenesis and exact mechanisms of neuronal destruction so more effective therapies can be investigated, ideally through robust randomized-controlled trials.

## Author Contributions

MG and AP drafted and revised the manuscript for intellectual content and direct patient care. Both authors contributed to the article and approved the submitted version.

## Conflict of Interest

The authors declare that the research was conducted in the absence of any commercial or financial relationships that could be construed as a potential conflict of interest.

## Publisher's Note

All claims expressed in this article are solely those of the authors and do not necessarily represent those of their affiliated organizations, or those of the publisher, the editors and the reviewers. Any product that may be evaluated in this article, or claim that may be made by its manufacturer, is not guaranteed or endorsed by the publisher.
